# The prognostic importance of duration of AKI: a systematic review and meta-analysis

**DOI:** 10.1186/s12882-018-0876-7

**Published:** 2018-04-19

**Authors:** Swati Mehta, Kinsuk Chauhan, Achint Patel, Shanti Patel, Rachel Pinotti, Girish N. Nadkarni, Chirag R. Parikh, Steven G. Coca

**Affiliations:** 1 Albany Medical center, 25 Hackett Blvd, Albany, NY 12208 USA; 2 Icahn School of Medicine at Mount Sinai, New York, NY USA; 3 Program of Applied Translational Research, Yale School of Medicine, Connecticut, NY USA

**Keywords:** Acute kidney injury, Duration, Mortality

## Abstract

**Background:**

Acute kidney injury (AKI), as defined by peak increase in serum creatinine, is independently associated with increased risk of mortality and length of stay. Studies have suggested that the duration of AKI may be an important additional or independent prognostic marker of increased mortality in patients with AKI across clinical settings. We performed a systematic review and meta-analysis of published studies to assess the impact of duration of AKI on outcomes.

**Methods:**

Various bibliographic databases (MEDLINE, Embase, Cochrane Library, CINAHL and Web of Science) were searched through database inception to December 2015. Human, longitudinal studies with patients aged 18 or above describing outcomes of duration of AKI were included. Duration of AKI categorized as “Short” if AKI duration was ≤2 days or labeled as “transient AKI”; “Medium” for AKI durations 3–6 days and “Long” for AKI duration of ≥7 days or “non-recovered”. Various outcomes looked at were Long term mortality, cardiovascular events, chronic kidney disease (CKD).

**Results:**

Eighteen studies were deemed eligible for the systematic review. The outcome of long-term mortality with duration of AKI was reported in 8 studies. The pooled Risk Ratio (RR) for long-term mortality generally was higher for longer duration of AKI: short duration of AKI (*n* = 8 studies, RR 1.42, 95% CI 1.21–1.66), medium duration (*n* = 4 studies, RR 1.92, 95% CI 1.34–2.75), and long duration (n = 8 studies, RR 2.28, 95% CI 1.77–2.94) duration of AKI. Further, Duration of AKI was independently associated with higher risk of cardiovascular outcomes and incident CKD Stage 3 when stratified within each stage of AKI.

**Conclusion:**

Duration of AKI was independently associated with long term mortality, cardiovascular(CV) events, and development of incident CKD Stage 3.

**Electronic supplementary material:**

The online version of this article (10.1186/s12882-018-0876-7) contains supplementary material, which is available to authorized users.

## Background

Acute kidney injury (AKI) is common in hospitalized patients and is independently associated with increased risk of morbidity, mortality and length of stay [1, 2]. AKI also leads to long-term kidney sequelae, such as chronic kidney disease (CKD), end stage renal disease (ESRD) and has long-term adverse cardiovascular effects [3, 4]. In the United States, AKI is one of the most serious and common health complications, occurring commonly in hospitalized patients and those in critical care settings [5, 6]. For these reasons, accurate risk stratification/classification of AKI and its effect on long-term complications are of prime importance.

Efforts have been made to define and classify AKI for use in clinical practice and research. Beginning with risk injury failure loss end stage (RIFLE) staging system, followed by the acute kidney injury network (AKIN), and most recently, the kidney disease improving global outcomes(KDIGO), these classification systems all assess the magnitude of serum creatinine elevation (change from baseline to peak creatinine) as the primary dimension to grade severity. AKIN and KDIGO do incorporate tempo for the rate of increase as a prerequisite (rise must occur over a period of 48 h) but none of the current classification systems take into account the duration of AKI, which reflects time to recovery and could be an additional important dimension of AKI severity.

Some studies have suggested that the duration of AKI may be an important additional or independent prognostic marker of long-term mortality, progression to CKD/ESRD and cardiovascular outcomes in patients with AKI in different clinical settings [7–15]. Furthermore, duration of AKI has also been suggested as a better endpoint for trials of AKI [16]. However, there has not been any systematic review or meta-analysis to quantify pooled estimates from these disparate studies. Therefore, we performed a systematic review and meta-analysis of the literature assessing impact of duration of AKI on survival, progression to CKD and cardiovascular outcomes.

## Methods

This study was performed in accordance with published guidelines for systematic review, analysis, and reporting for meta-analysis of observational studies [17].

### Inclusion and exclusion criteria

Studies were included if they provided information about duration of acute kidney injury in hospitalized patients. The settings for hospitalization included: 1. Medical non intensive care; 2. surgical non intensive care-not post-surgical; 3. Medical-intensive care; 4. surgical intensive care-not post-surgical; 5. surgical intensive care-post surgical-cardiac surgery and 6. Surgical intensive care-post surgical-non cardiac surgery and reported at least one of the outcomes: short or long term mortality, cardiovascular outcomes and progression to chronic kidney disease (CKD) or end stage renal disease (ESRD). Observational studies (prospective cohort (PC) / retrospective cohort (RC) / evaluating human adult patients with age ≥ 18 years admitted to the hospital with AKI were included. There was no publication year restriction. We excluded in vitro / in vivo studies, case reports / case series, narrative reviews, book abstracts, editorials / authors’ replies, and systematic reviews / meta-analyses (after scanning references for relevant articles) and non-English articles.

### Literature review and study selection

A comprehensive search query employing a combination of appropriate subject headings and keywords was developed in collaboration with a qualified medical librarian. The search was executed in the MEDLINE, Embase, Cochrane Library, CINAHL and Web of Science databases from database inception through December 2015. Animal studies were excluded using the search statement recommended by the Cochrane Collaboration. The full electronic search strategy for all databases is available in Additional file 1. An additional 123 citations, identified using PubMed’s Similar Articles algorithm and after reviewing the references of included articles to identify additional articles, were included in the screening library.

Search results were independently screened by two reviewers (SM, SP). In the case of disagreement among reviewers, a third reviewer (GN) arbitrated. Study selection occurred in two successive rounds, the first round based on titles and abstracts, the second round based on full text.

### Data collection

Two review authors (SM,SP) independently extracted relevant study characteristics and outcomes from the included studies using a standardized and piloted data extraction form. The information extracted from each study was: author, year of publication, characteristics of participants (number, age and sex), clinical setting, type of study (prospective vs retrospective), study period and follow up, definition and severity of AKI, duration of AKI and incidence of outcomes (Long term mortality, cardiovascular events and progression to CKD and ESRD) amongst participants with different duration of AKI. AKI was ascertained either by using the standard AKIN/KDIGO/ RIFLE criteria or AKI (increase in serum Creatinine of at least 25–30% of baseline) vs no AKI and ATN (Sudden rise in serum Creatinine to more than 2 mg/dl in subjects with prior normal renal function) vs no ATN. Due to significant heterogeneity of definitions of duration of AKI, we created the following 3 categories of duration of AKI: “Short” if AKI duration was ≤2 days or labeled as “transient AKI”; “Medium” for AKI durations 3–6 days and “Long” for AKI duration of ≥7 days or “persistent /non-recovered”. Any discrepancies about date extraction were resolved by a third reviewer (GN). The study was conducted through August 2015 to June 2016.

### Quality assessment

Two independent reviewers assessed study quality according to criteria outlined by Downs and Black using the 27-point checklist included within 5 main sections [18]. The five sections include questions about: 1. Reporting (10 items); 2. External validity (3 items); 3. Internal validity/Bias (7 items); 4. Confounding (selection bias) (6 items); and 5. Power of the study (1 item).

Studies were graded as good quality if scored 20 or higher, fair quality if scored 15 or higher and poor quality with below 14 scores in the checklist. Please refer to Additional file 2 for details.

### Outcome measures

Primary outcome measures were the adjusted risk ratio (RR) for long-term mortality associated with AKI duration. We also aimed to compare effect sizes of associations to standard AKIN/KDIGO Stages. Secondary outcomes were the risk for cardiovascular outcomes and incident CKD. In two of the studies with cardiovascular outcomes - the cardiovascular events were de novo, [10, 19] while one study included pts. with prior cardiac disease and then aimed at determining the association of duration of AKI and risk for CV events beginning 90 days after discharge [11]. We also assessed the adjusted RR for cardiovascular outcomes and incident CKD stage 3 with duration of AKI when stratified within each stage of AKI.

### Statistical analysis

Random effects meta-analysis was conducted to estimate the magnitude of risk associated with duration of AKI for each outcome, as measured by combined adjusted RR with 95% confidence intervals. Adjusted risk estimates included those published in final multivariate models for each study, which considered confounding from sociodemographic and clinical covariates, such as age, gender, race, co-morbidities, medications, and laboratory values. Adjusted pooled risk ratios for duration of AKI were estimated with inverse variance method using Review Manager 5.3. We formally assessed heterogeneity of effects between studies with the I^2^ Statistic.

## Results

**Fig. 1:**
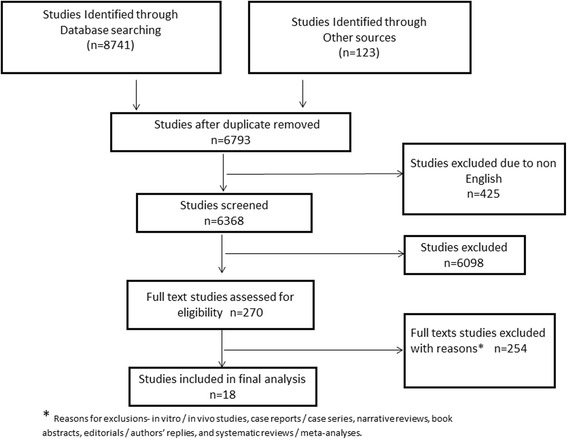
Prisma flow diagram of studies that were considered for inclusion

We identified 8864 studies meeting our search criteria. After excluding 2071 duplicate and 425 non-English studies, 6368 were evaluated, and 270 were selected for full text review. After application of exclusion criteria 18 studies were deemed eligible for this systematic review (Fig. 1).

Study and patient characteristics from the selected articles are summarized in Table 1. These 18 studies were comprised of 459,558 patients with length of follow-up-median,3 years; IQR,1.8 to 4.75 years. Baseline Cr definition varied-Most studies defined baseline Cr as value recorded just prior to surgery or hospitalization, 1 study [20] defined it as- mean of all outpatient Cr in 6 months before hospitalization, 1 study [19] as Serum Cr less than 1.4 mg/dl.

**Table 1:** Characteristics of studies included in the systematic review and meta-analysis

First Author	Year	Single vs. Multi Center	Type of Study	Clinical Setting	N	Years of Follow Up		Age Range	Percent Male	Length of Hospital Stay in Days^a^	AKI definition severity	Duration of AKI		Study Quality	Different covariates adjusted for
						Mean	Max					Short, medium or long	Transient or Persistent		
Brown	2010	Single	R	Cardiac surgery	4987	2.6	5	64–72	29–33	6–24.	AKIN	√		Fair	age, sex, prior CABG, COPD, emergency surgery, ejection fraction and baseline eGFR
Coca [7]	2010	Multi	R	Non cardiac	35,302	3.8	9	66–88	96–98	6	AKIN	√		Good	age, sex, race, chronic insulin use, operative time, ASA class 4/5, emergency surgery, baseline GFR, smoking status,weight loss > 10% last 6 months, chronic alcohol intake, COPD, pre-op albumin, hematocrit,WBC, and hemoglobin A1C
Choi [11]	2010	Multi	R	General Hospital	17,325	5.7	20	44–47	98	NM	AKIN		√	Fair	Age, sex, race, baseline GFR, albuminuria, viral load, cd4, htn, dm, lung ds, smoking, cancer, ICU admission
Goldberg [19]	2009	Single	R	Post MI	1957	1.5	5	59–70	76	NM	AKIN		√	Fair	age, gender, estimated GFR (MDRD), previous diuretic therapy, HTN,DM, smoking, previou MI, heart rate and blood pressure on admission, use of reperfusion therapy ACE/ARB and beta-blockers), and left ventricular EF.,
Han [12]	2013	Single	R	ICU	2143	0.4	0.5	68	52–68	13–29	KDIGO	√		Fair	Age, sex, Apache, primary diagnosis, underlying CKD, history of malignancy, the need for mechanical ventilation, the use of vasoactive drugs, and AKI stages
Loef [26]	2004	Single	R	Cardiac surgery	843		8.3	62–64	73	1–5	AKI/ No AKI			Fair	Age, PVD,Operation time, pre op renal function by cockroft-gault formula,post op renal function deterioration
TRIBE-AKI [21]	2016	Multi	P	Cardiac surgery	1199	3		71–73	66–76	7–32	AKIN	√		Good	Age, sex, race, elective surgery, preop GFR, DM, HTN, CHF, MI,surgery type and center
Wu [14]	2015	Single	R	Intensive	318	8		65.6	71	31	KDIGO	√		Good	age, gender, need for a ventilator, emergent operation and baseline renal function; severity of sepsis, the use of vasoactive drugs and diuretics, surgery category; APACHE II, MODS, SOFA scores and peak KDIGO stage.
Yoo [13]	2014	Single	P	General Hospital	123	0.65	3.4	64.8	61	39	AKIN	√		Good	Age, sex, charlson comorbity index, recovery
Pannu [20]	2013	Multi	R	General Hospital	190,714	2.8	6	63–66	47–53	NM	KDIGO		√	Good	age, sex, MI, PVD,CVA,CHF,DM, nondermatologic malignancy, baseline estimatedGFR,requirement for acute dialysis, primary diagnostic code for hospitalization, CIHI resource intensity weight.
Liano [19]	2006	Single	R	General Hospital	413	7.2	22	57.8	66	54	ATN/ No ATN		√	Poor	Age, sex, type of admission, ATN etiology, need for RRT, ARF functional severity, ICU admission,incidence of comorbity factors
Uchino [27]	2010	Single	R	General Hospital	20,126		2	65–78	49–56	NM	RIFLE		√	Fair	Age, sex, emergency admission, ICU admission,mech ventilation,baseline Cr in mg/dl,operation time
Wamock [28]	2015	Single	R	General Hospital	50,580	28		55	55–63	4–8	AKI/ No AKI		√	Fair	Age, race, sex, charlson comorbid index, admission source, egfr < 60
Gammelager [10]	2014	Multi	R	ICU	21,550	2.7	3	57–68	53–61	8–23	KDIGO		√	Fair	age, gender, other ischemic heart diseases, CVA,HTN, PVD, CKD cancer, surgical status, primary diagnosis during current hospitalization, and preadmission use of drugs
Welton [29]	2007	Single	R	Cardiac surgery	1324	6	10	66	80	NM	AKI/ No AKI		√	Good	Age, sex, HTN, DM, smoking,Hl, COPD, BMI,Prior MI and coronary revascularization,angina, CHF, baseline Cr Clearance, medications, and short term complications.
Heung [9]	2015	Single	R	General Hospital	104,764		13	61.8	95	7	KDIGO	√		Good	Age, race, sex, preadmission,DM, HTN,Dx of sepsis, need for mechanical ventilation during index hospitalization, length of stay,charlson comorbity score and baseline GFR.
Sood [30]	2014	multi	R	ICU	5443	0	0	55–70	57	NM	RIFLE		√	Good	Demographics, illness severity, co-morbidity,and treatment
Perinel [31]	2015	multi	R	ICU	447	0	0	45–75	63.3	NM	AKIN		√	Good	Age, type of AKI, use of vasopressors, illness severity

*R* Retrospective, *P* Prospective, *AKIN* Acute Kidney Injury Network, *HIV* Human Immunodeficiency Virus, *NM* Not Mentioned, *ICU*- Intensive Care Unit, *KDIGO* Kidney Disease: Improving Global Outcomes, *ATN* Acute Tubular Necrosis, *RIFLE* Risk, Injury, Failure, Loss of kidney function, and End-stage kidney disease classification, *AMI* Acute myocardial infarction, *DM* Diabetes Mellitus, *HTN* Hypertension, *PVD* Peripheral vascular Disease, *CHF* Congestive heart failure, *MI* Myocardial infarction, *BMI* Body mass index, *CVA* Cerebrovascular accident, *Dx* Diagnosis, *Cr* Creatinine

^a^Median days of length of hospitalization as reported by authors

Duration of AKI was defined as either short (≤ 2 days), medium (3–6 days) or long (≥ 7 days)or transient(renal recovery at hospital discharge with Cr returning to either baseline or atleast within 25–50% of baseline without requirement of renal replacement therapy during hospitalization) and persistent/non recovered(no renal recovery at hospital discharge and not meeting above criteria). 7 out of 16 studies categorized duration of AKI as short, medium or long and the remaining 11 studies categorized AKI as transient vs. persistent. 17 out of 18 studies met the quality criteria, were fair to good quality (Table 1 and Additional file 2). The definitions of AKI severity varied substantially (Table 1) 14 out of 18 studies defined AKI using standard AKIN/RIFLE and KDIGO definitions. 3 studies defined AKI as increase in serum Creatinine of at least 25–30% of baseline and the remaining one study used AKI as synonymous with ATN defining it as sudden rise in serum Creatinine to more than 2 mg/dl in subjects with prior normal renal function**.** The overall incidence of AKI was 15%, and of those with AKI in the 7 studies that stratified duration as short, medium and long, 68% experienced short duration, 17% medium duration, and 13% long duration AKI. Then for remaining 11 studies, 60% experienced transient and 40% experienced persistent or non-recovered AKI. 8 out of 18 studies had approximately 5.32% subjects requiring renal replacement therapy amongst long duration or persistent AKI. We then grouped transient with short duration of AKI and persistent or non-recovered AKI with long duration of AKI in order to align participants by duration of AKI. The most common clinical setting for the studies of AKI duration was general hospitalized patients (*n* = 6), cardiac surgery (*n* = 5) and critical illness (n = 5). Most studies were single center (*n* = 11) and retrospective (*n* = 16).

### Duration of AKI and long-term mortality

**Fig. 2:**
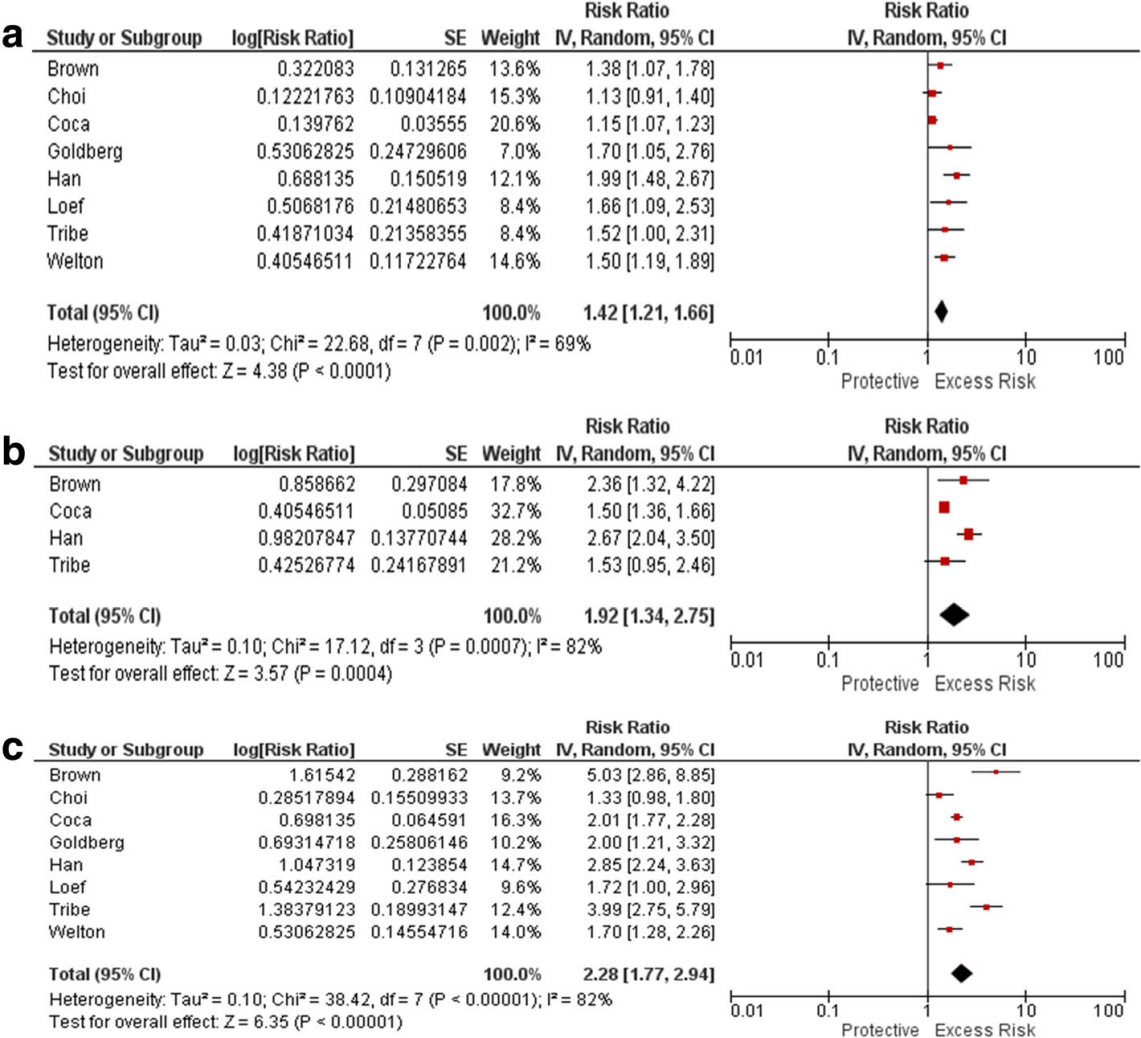
Pooled RRs for long term mortality by short (**a**), medium (**b**) and long (**c**) duration of AKI

The outcomes of long-term mortality with duration of AKI were reported in 8 studies. Three of the eight studies [7, 8, 21], reported risk for mortality for duration of AKI stratified within each stage of AKI 1, 2 and 3, as defined by peak changes in creatinine. Long term mortality generally was higher for longer duration of AKI: short duration of AKI (*n* = 8 studies, RR 1.42, 95% CI 1.21–1.66; Fig. 2a), medium duration (*n* = 4 studies, RR 1.92, 95% CI 1.34–2.75; Fig. 2b), and long duration (n = 8 studies, RR 2.28, 95% CI 1.77–2.94; Fig. 2c) of AKI. There was considerable heterogeneity across pooled studies.

We also made separate forest plots for short, medium and long duration and transient(recovered) and persistent (non recovered) AKI. Even with separate forest plots, the pooled Risk Ratio (RR) for long-term mortality generally was higher for longer duration of AKI or Persistent (non recovered) AKI. Please refer to Additional files 3 and 4 respectively.

### Duration of AKI and association with long-term cardiovascular outcomes

**Fig. 3:**
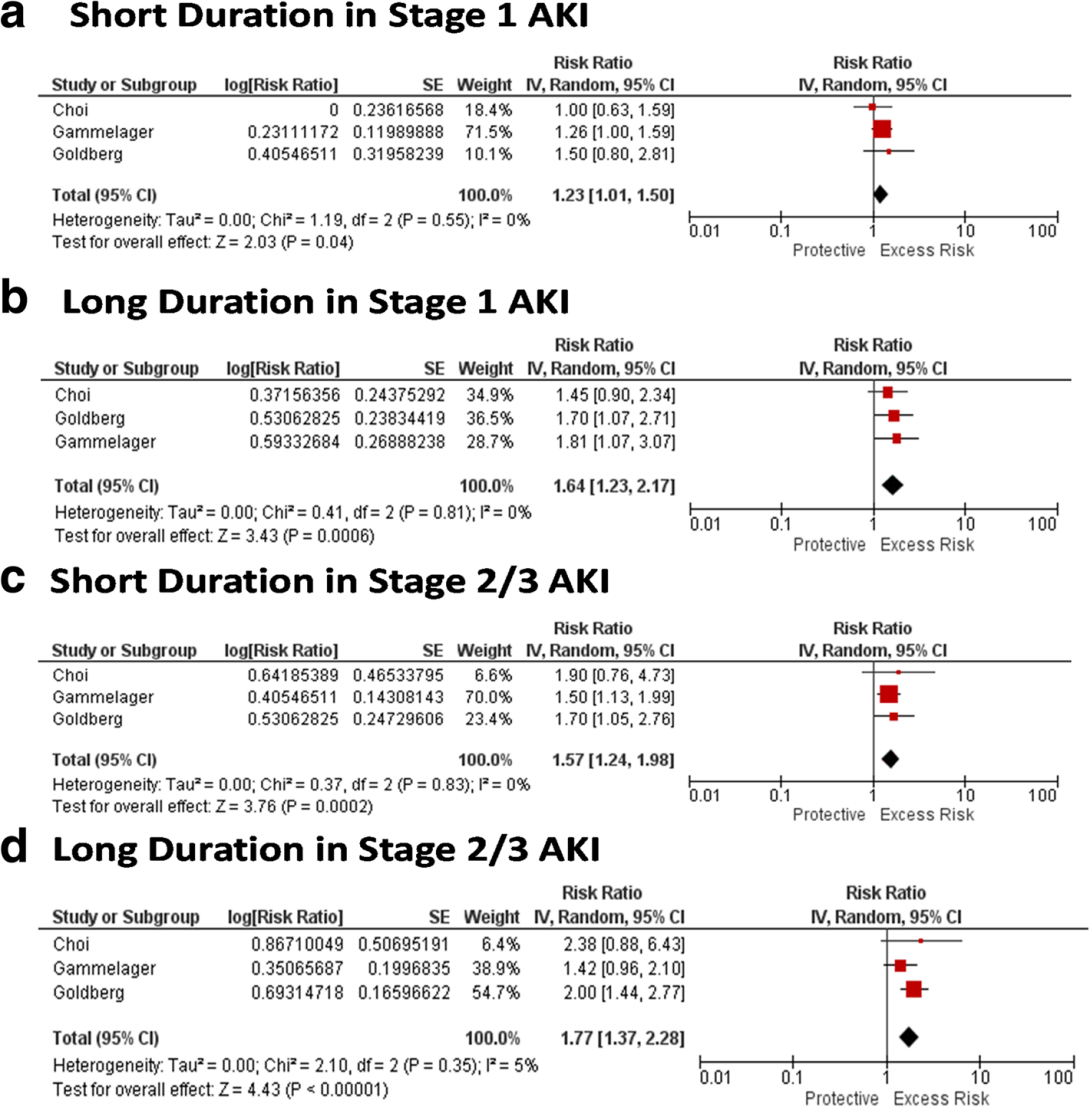
RR for CHF by duration within strata of AKI

**Fig. 4:**
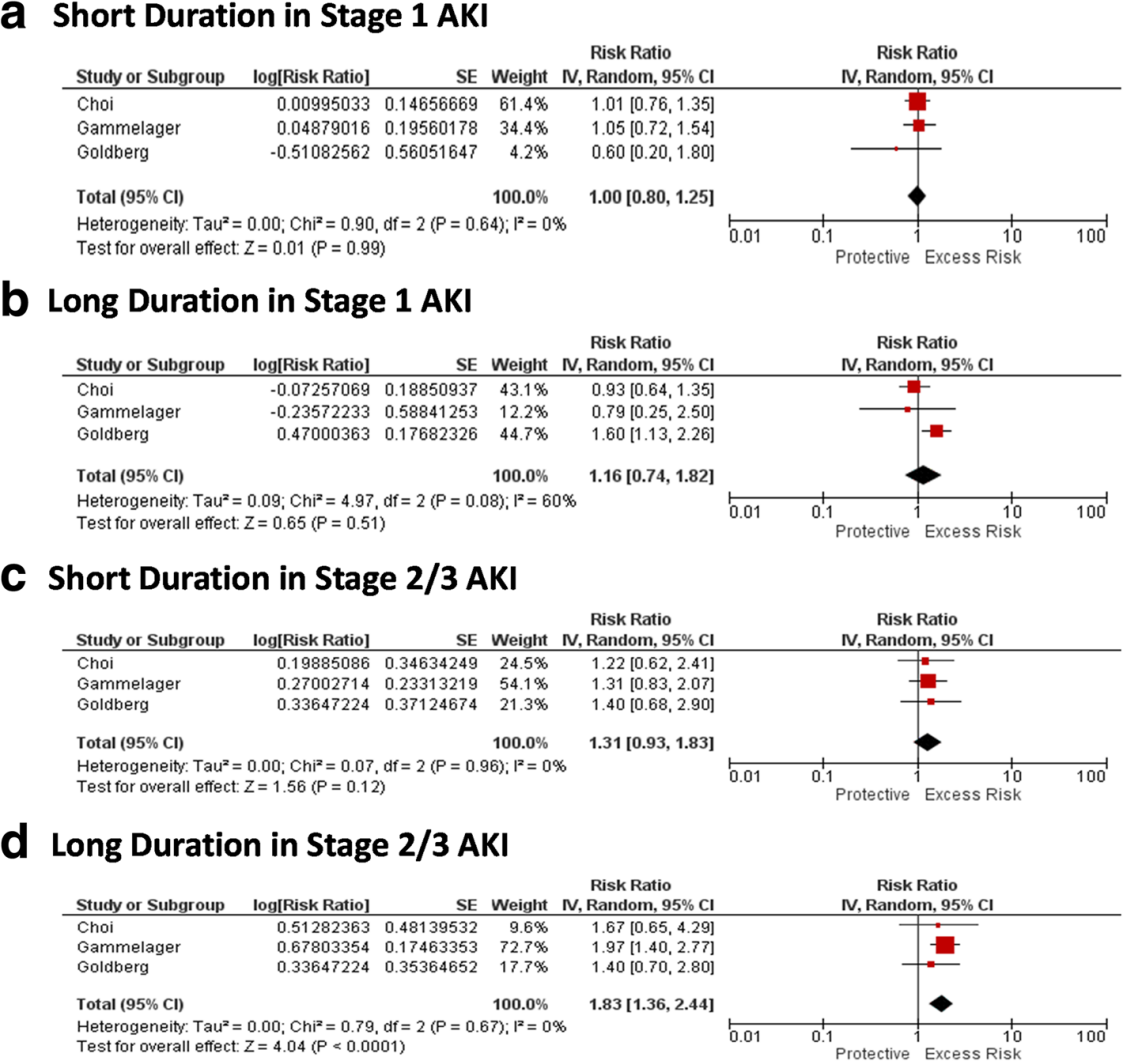
RR for MI by duration within strata of AKI

Three studies reported on cardiovascular outcomes, including congestive heart failure (CHF) and myocardial infarction (MI), in association with differing durations of AKI. Duration of AKI added additional prognostic value to AKIN stages of AKI for the outcome of CHF (Fig. 3a, b, c, d) and duration of AKI added additional prognostic value in patients with stage 2 or 3 AKI for the outcome of MI, but not for those with stage 1 AKI (Fig. 4a, b, c  and d).

### Duration of AKI and incident CKD

**Fig. 5:**
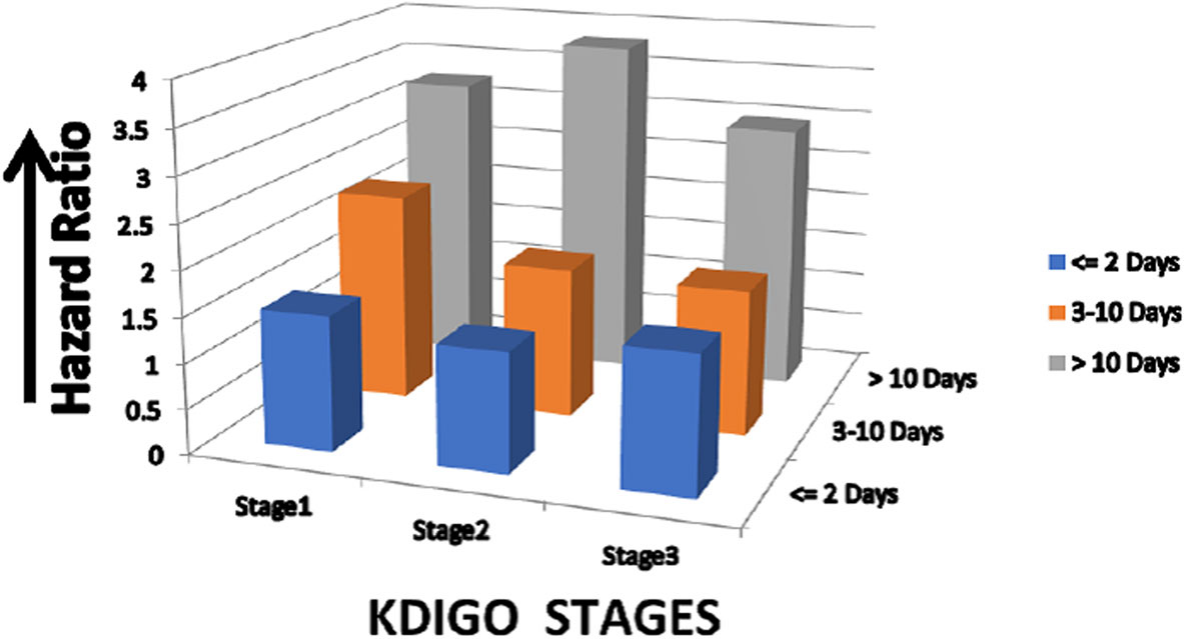
Risk for incident CKD stage 3 increases with duration even after adjusting for KDIGO stage

Two studies reported on the risk for CKD in association with the duration of AKI. Heung et al. examined 104,764 veterans with baseline eGFR > 60 mL/min/1.73 m^2^ and demonstrated a dose-response relationship between the duration of AKI and the risk for development of CKD stage 3 stratified across all AKI stages (Fig. 5) [9]. Palomba et al. examined the risk for CKD after cardiac surgery, and found that those with AKI duration > 3 days had an adjusted OR for incident CKD of 13.5 (95% CI 4.2–43.7) [22].

## Discussion

This is the first systematic review and meta-analysis to date that summarizes the association of duration of AKI with several important outcomes. While there was heterogeneity across studies due to different study design and definitions of AKI and schema of classifying duration, the results suggest consistently and strongly that there is a dose-response relationship between duration of AKI and long-term outcomes, including death, cardiovascular outcomes, and incident CKD.

The current consensus definitions of AKI as proposed by AKIN/KDIGO or RIFLE do not incorporate any duration component into the definitions. However, based on the available data summarized in this manuscript, it appears that the duration of AKI provides additive risk information for long-term outcomes among survivors after hospitalization with AKI. While few studies accounted for the severity of AKI by existing definitions and assessed the additional independent contribution of duration, there was a strong independent signal for outcomes, particularly in the studies that assessed CV events and incident CKD. Our study emphasizes that patients with AKI stages 2 and 3 are at increased risk of CHF and MI, however, the risk is more pronounced in patients without recovery of renal function. This may suggest a need for systematic follow up of patients discharged after an AKI episode, especially those who did not regain their renal function before discharge [9–11, 23].

Based on the above findings of our systematic review and meta-analysis, we propose that the duration of AKI should be incorporated into future schemas for classification of AKI for prognosis of long-term outcomes. Most of the studies on long-term prognosis after AKI mostly focus on characterizing the severity of AKI by magnitude of rise in serum creatinine, but hopefully this systematic review will encourage clinical researchers and epidemiologists to use at least two-dimensional approaches (magnitude of serum creatinine rise and duration of AKI) to evaluate outcomes of AKI for future investigations. However, recently long-duration of AKI / persistent AKI is equivalent to the new term of Acute kidney disease(AKD). AKD is a newly recognized term by the Acute dialysis quality initiative (ADQI) in their consensus report [24] that recognizes the important consequences of AKI duration of 7 days or more (ARD).

This review also focuses attention on the fact that a patient who experiences a large but transient increase in serum creatinine is prognostically different from patient who experiences a sustained increase in serum creatinine. While the current classification of AKI assigns higher risk to the first patient given the rise in creatinine is numerically higher, the findings suggest that the second patient has equal or higher risk of worse outcomes. Further research is needed as how to effectively treat such patients and whether close follow up of these patient’s post- hospital discharge improves outcomes.

Duration of AKI could be similar to or atleast an overlap to the concept of “kidney recovery” at hospital discharge. Recently, Kellum et al. [25] characterized various patterns of kidney recovery after an episode of AKI and related these patterns to long term outcomes. They found that early reversal was associated with the shortest ICU stay and best prognosis, 1-year survival, 90.2% and those who never recovered or had reversal with relapse had the worst prognosis, 1-year survival, 40%. These results are similar to our findings of longer duration of AKI with worse long-term mortality.

The findings herein have additional implications with regards to clinical trials. Using mathematical modeling of creatinine changes in AKI, it has been suggested that incorporating time to creatinine elevation into AKI definitions may be a more efficient and a less biased way of ascertainment of outcomes in interventional trials for AKI [16]. Therefore, one could propose that AKI duration should be considered to improve the endpoints for phase 2 trials of new agents instead of just incident AKI by peak creatinine as an outcome. The downside of using incident AKI as an outcome is that an investigational drug/therapy will have to work at a particular time point or earlier (for example in pre-renal phase of Acute Tubular Necrosis, ATN) to reduce AKI incidence. In contrast, if duration of AKI is employed as an outcome, one can show the intervention to be effective if it works on any of the phases of ATN (initiation, propagation, maintenance, and recovery phases), thereby decreasing duration of AKI. Moreover, long duration of AKI might serve as an enrollment criterion for interventions that aim to abrogate the transition from AKI to CKD.

### Strengths and limitations

The strengths of this analysis include adequate length of follow up in the studies which looked at long term mortality, cardiovascular outcomes and incident CKD stage 3 and rigorous study selection and pooling of only adjusted estimates. Also, the results of the study could be generalized to various clinical settings and to varying populations. Despite above, our study should be interpreted in light of some limitations. Although, we performed an exhaustive search of the literature for duration of AKI studies, publication bias cannot be ruled out. It is possible that smaller negative studies were not published. We considered formally assessing publication bias through a funnel plot; however, funnel plots cannot be interpreted in the presence of marked heterogeneity. Another important limitation is the heterogeneous definition of duration of AKI used in different studies enabling us to group transient with short duration of AKI and persistent or non-recovered AKI with long duration of AKI in order to align participants by duration of AKI. However, this resulted in a high degree of statistical heterogeneity. Also, there were only two studies published that associated AKI duration with subsequent CKD and there is lack of data on the outcome of ESRD. As for most of the meta-analysis, our meta-analysis is also limited by the quality of the primary studies. Only 7 studies were graded as good quality with the primary limitation in other studies being the internal validity-bias (Additional file 2). The association between duration of AKI and outcomes is also highly prone to be a surrogate for severity of illness. While the studies adjusted for known confounders, certainly residual confounding was present and could not be eliminated, thus potentially biasing the point estimates away from null. Lastly, the population in the study was mostly male, whether the same findings apply to females is unknown and should be the subject of further investigation.

## Conclusion

In conclusion, duration of AKI is independently associated with long-term mortality and may provide additional prognostic information over and above magnitude of serum creatinine alone. Thus, AKI duration can be considered as a prognostic factor for long-term mortality and other cardiovascular outcomes and can be used as an endpoint in intervention trials to prevent or treat AKI.

## Additional files

Additional file 1: Systematic review search strategy. (DOC 24 kb)

Additional file 2: Quality Assessment. (XLSX 17 kb)

Additional file 3: Separate forest plot for long term mortality with short, medium and long duration of AKI. (TIFF 206 kb)

Additional file 4: Separate forest plot for long term mortality with transient and persistent duration of AKI. (TIFF 72 kb)

## Abbreviations

ADQI: Acute dialysis quality initiative
AKD: Acute Kidney disease
AKI: Acute kidney injury
AKIN: Acute kidney injury network
ATN: Acute tubular necrosis
CKD: Chronic Kidney disease
CS: Cross sectional
CV: Cardiovascular
ESRD: End stage renal disease
KDIGO: Kidney disease improving global outcomes
PC: Prospective cohort
RC: Retrospective cohort
RIFLE: Risk injury failure loss end stage
RR: Risk ratio
