# Supplementary material for: The prognostic importance of duration of AKI: a systematic review and meta-analysis
Source: BMC Nephrol. 2018 Apr 19;19:91. doi: 10.1186/s12882-018-0876-7 (PMC5907696; doi:10.1186/s12882-018-0876-7)
Supplement: Supplementary file 1 — Systematic review search strategy. (DOC 24 kb) [file 12882_2018_876_MOESM1_ESM.doc]

# Additional file 1

Systematic Review Search Strategy

**MEDLINE (PubMed Platform)**

((((("Acute Kidney Injury"[Mesh]) AND (((acute) AND (kidney OR renal)) AND (injur* OR insufficienc* OR failure*)))) AND (("Time Factors"[Mesh]) OR duration*))) NOT ((animals[MeSH Terms]) NOT ((animals[MeSH Terms]) AND humans[MeSH Terms]))

**Embase (Ovid Platform)**

1. acute kidney failure/

2. (acute adj3 (kidney or renal) adj3 (injur* or insufficienc* or failure*)).mp. [mp=title, abstract, heading word, drug trade name, original title, device manufacturer, drug manufacturer, device trade name, keyword]

3. 1 or 2

4. disease duration/

5. time/

6. duration*.tw.

7. 4 or 5 or 6

8. 3 and 7

9. animal/

10. human/

11. 9 not (9 and 10)

12. 8 not 11

**Cochrane Library (Ovid Platform)**

1. exp Acute Kidney Injury/

2. (acute adj3 (kidney or renal) adj3 (injur* or insufficienc* or failure*)).mp. [mp=ti, ot, ab, sh, hw, kw, tx, ct]

3. 1 or 2

4. time factors/

5. duration*.tw.

6. 4 or 5

7. 3 and 6

**CINAHL (Ebsco Platform)**

S4 OR S5

duration OR transient OR persistent OR tertile OR time factor*

(MH "Disease Duration")

S1 OR S2

acute AND ( (kidney OR renal) ) AND ( (injur* OR insufficienc* OR failure*) )

(MH "Kidney Failure, Acute")

**Web of Science**

TOPIC: (acute near/3 (kidney OR renal) near/3 (injur* OR insufficienc* OR failure*)) ANDTOPIC: (duration*) NOT TOPIC :(animal NOT (animal AND human))

Timespan: All years. Indexes: SCI-EXPANDED, SSCI, A&HCI, ESCI
