# Supplementary figures and images for: The prognostic importance of duration of AKI: a systematic review and meta-analysis
Source: BMC Nephrol. 2018 Apr 19;19:91. doi: 10.1186/s12882-018-0876-7 (PMC5907696; doi:10.1186/s12882-018-0876-7)

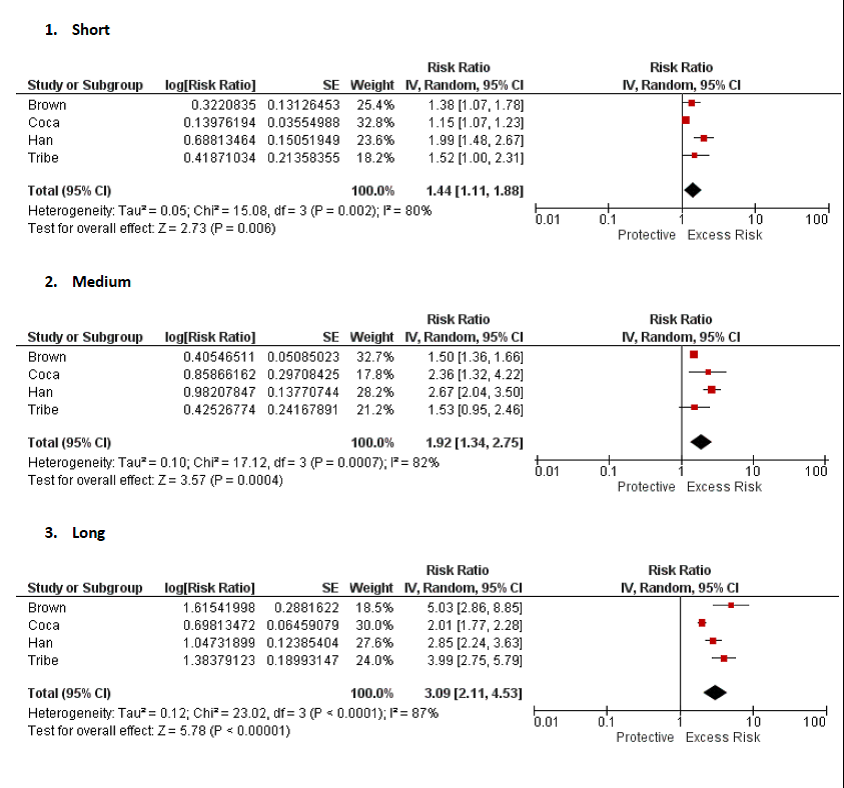

Supplement: Supplementary file 3 — Separate forest plot for long term mortality with short, medium and long duration of AKI. (TIFF 206 kb) [file 12882_2018_876_MOESM3_ESM.tif]

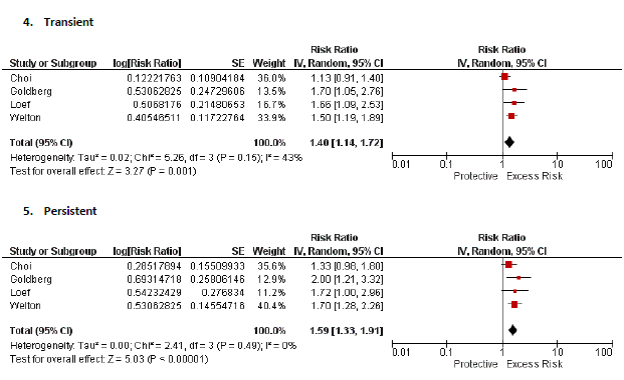

Supplement: Supplementary file 4 — Separate forest plot for long term mortality with transient and persistent duration of AKI. (TIFF 72 kb) [file 12882_2018_876_MOESM4_ESM.tif]
